# Effect of TGF-β3 on wound healing of bone cell monolayer in static and hydrodynamic shear stress conditions

**DOI:** 10.3389/fmed.2024.1328466

**Published:** 2024-04-24

**Authors:** Hawra Al-Attar, Laila A. Damiati, Saeed Heidari Keshel, Cristina Tuinea-Bobe, Samar Damiati, Morvarid Saeinasab, Farshid Sefat

**Affiliations:** ^1^Department of Biomedical and Electronics Engineering, School of Engineering, University of Bradford, Bradford, United Kingdom; ^2^Department of Biological Sciences, Collage of Science, University of Jeddah, Jeddah, Saudi Arabia; ^3^Department of Tissue Engineering and Applied Cell Sciences, School of Advanced Technologies in Medicine, Shahid Beheshti University of Medical Sciences, Tehran, Iran; ^4^Interdisciplinary Research Centre in Polymer Science & Technology (Polymer IRC), University of Bradford, Bradford, United Kingdom; ^5^Department of Chemistry, College of Sciences, University of Sharjah, Sharjah, United Arab Emirates; ^6^Faculty of Biology, Medicine and Health, University of Manchester, Manchester, United Kingdom

**Keywords:** wound healing, transforming growth factor beta, bone cell engineering, static conditions, dynamic conditions

## Abstract

**Introduction:**

Wound healing is characterized as a complicated and sophisticated biological process through which tissue heals and repairs itself after injury. However, the normal wound healing process relies on different growth factors as well as the presence of an accurate cytokine level to ensure appropriate cellular responses. In the case of wound healing, the effects of various growth factors have been studied, but the effects of transforming growth factor beta (TGF-β) on wound healing have been found to be more significant because of its broad spectrum of impacts on healing the wounded tissues or skins.

**Methods:**

In the current study, the impact of TGF-β3 in bone cells’ wound healing was examined *in vitro*. Furthermore, the activities and characteristics of TGF-β3, as well as those of related growth factors throughout this wound healing process, were studied under hydrodynamic shear stress conditions as well as static conditions of cultured bone cells.

**Results:**

We demonstrated that a positive outcome of TGF-β3 treatment was found after 24 h under a static condition, while TGF-β3 treatment was found to be effective under a dynamic condition for wound closure. In the case of the dynamic condition, a full wound closure was obtained after 18 h in both the control and TGF-β3 treatment, while in the case of static conditions, wounds were found to remain open, even after 24 h, for both the control and TGF-β3 treatment. Additionally, in the static condition, the wound closure rate with TGF-β3 treatment was found to be quicker than that of the control flask, which implies that wound healing can be postponed in the static condition. In the dynamic condition, the wound healing process became more rapid in a cultured cell environment.

**Conclusion:**

The synergistic effect of TGF-β3 and hydrodynamic shear stress conditions had a positive impact on increasing wound healing and improving the rate of wound closure.

## GRAPHICAL ABSTRACT

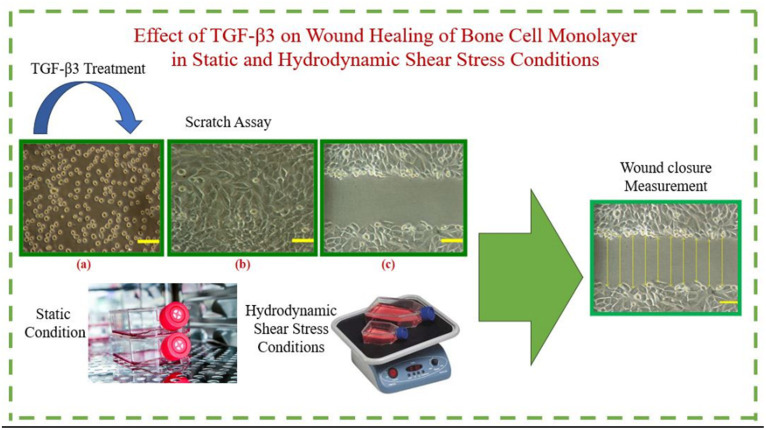

## 1 Introduction

Wound healing is a complex biological process that involves four different stages: hemostasis, inflammation, proliferation, and remodeling of the wounded tissue. The wound healing process is completed with the coordination and integration of all these steps (1). This process is typically modulated systematically (by estrogens and calcitonin) and locally (by cytokines and the presence of growth factors), and there is always an integration between these factors so that they can interconnect with each other to achieve hemostasis of the bone (2). Bone is the most important component of the human body, providing the structure of the human body. Bone formation is a function coordinated by numerous cells including osteoblasts, osteocytes, osteoclasts, and osteoprogenitors (3). It is well established that bone tissue has its own blood supply within the bone structure and, therefore, the bone healing process for wounds or fractured bones is initiated by blood release from the bone tissues that assesses the targeted wound and starts coating the fracture by blood coagulation. When a soft layer of collagen begins to line the wound, the bone cells require more time to build the bone and become rigid. There are different factors which are associated with the wound healing process and those factors may facilitate or hinder the process. An intricate biological process involving cell membrane and cellular interaction is the fundamental concept of bone healing. Bone healing consists of various aspects that play a crucial role in wound healing, such as the involvement of growth factors (TGF-β) and other cellular components in the regeneration process (4). Cells within human tissues create cytokines, one of the most important biomolecules in the human body, and most cell functions are normally regulated by cytokines. Furthermore, cytokines play a significant role in the introduction and prevention of different diseases in the human body (5). One of the most common cytokines is TGF-β, created by the cells in human tissues. There are three common isomers of TGF-β: TGF-β1, TGF-β2, and TGF-β3. These growth factors are involved in various cellular functions such as cell relocation, multiplication of cells, apoptosis, and cell attachment. All these isomers are involved in the control process for cellular development and improvement. However, the functions of various TGF-β isomers differ with respect to the site of the target cells as well as due to the variation of environments (6). TGF-β also plays a vital role in inducing the regeneration process of bones; however, the ability of TGF-β to repair the fractured bone in bone wound healing and bone regeneration depends on the mechanical stability of the fracture in the bone at the target site (7).

The underlying mechanism of wound healing for bone cells was investigated *in vitro* in this study, with a particular focus on the growth factors (TGF-β3). The behavior of TGF-β3 in bone cells’ wound healing was examined through the wound closure rate analysis of the cultured bone cell monolayers in both the dynamic condition in the presence of hydrodynamic shear stress and in the static condition. This research work in a continuation of previous research which investigated the effect of various TGF-β isomers including TGF-β1,2,3, and combination of them (8, 9). The main aim of this project is to investigate the hydrodynamic shear stress condition that was not investigated previously neither by our team nor other research teams.

## 2 Materials and methods

### 2.1 Cell culture

MG-63 osteosarcoma bone cells (Sigma-Aldrich, UK) were cultured in Dulbecco’s Modified Eagle Media (DMEM) containing low glucose (1 g/L-D-glucose) with a 25 mM HEPES, 10% (v/v) fetal bovine serum (FBS), penicillin (100 U/mL), streptomycin (0.1 mg/mL), and fungizone (250 μg/mL) (Sigma–Aldrich, UK). The bone cells were cultured into 25 cm^2^ tissue culture flasks, at cell density of 4 × 10^5^ cells/flask. For the dynamic condition, the flasks were placed into the incubator at 37°C in 5% CO_2_ and configured at 50 rpm (Rocker machine), while other flasks were kept at BSA static condition.

### 2.2 Reconstitutions

TGF-β3 (Sigma-Aldrich, UK) was diluted, according to supplier recommendation, with HCl and BSA. 10 mg BSA was dissolved in 10 mL of 4 mM HCl to obtain 1 mg/mL HCl/BSA. Following this, the solution was sterilized, using a 0.22 μm filter. 2 μg of TGF-β3 was dissolved in 0.4 ml of 1 mg/mL HCl/BSA to give a working concentration of 50 ng/mL and aliquoted into forty 10 μl vials and stored at −20°C. All culture flasks were treated with 50 ng/ml TGF-β3 by adding the exact dose to 5 mL of media immediately after creation of wound. This dose was obtained and optimized by previous work that compared and investigated the effect of various TGF-β isomers including TGF-β1,2,3, and combination of them (8, 9).

### 2.3 Wound creation on cell monolayers

MG-63 bone cells were refined and mixed, and in this process, the cell monolayers were scratched with the tip of a 1 mm plastic tube. The tip of the tube was placed downwards so that it could be embedded into the flask, and then it was drawn over the cells to make wounds on the cells. Then, the underside of the flask was set apart with three parallel lines, with the help of permanent marker, in such a way that the lines remained parallel to the wounds attached to the cell monolayers. A test was conducted to ensure the time required for wound closure was 48 h. This was similar to another test for determining wound closure of MG-63 bone cells in which wound closure was achieved after approximately 30 h (8). After scratching the cell monolayer, around 300 μm of wound was created, though there were some deviations (Supplementary Figure S1). To standardize the wound width, mean value was measured for each flask; each starting wound width was considered as the rate of that starting wound width. The test was intended to be completed within 24 h; however, for the static condition, complete wound closure was not completed after this period. Therefore, data were recorded for 48 h, at 2-h intervals, to observe the wound closure at static condition. Eight culture flasks were studied with wounded monolayer bone cells and, of these, two culture flasks were treated with TGF-β3 and another two flasks were considered as control without any treatment. Data were recorded for 48 h, at 2-h intervals, to observe the wound closure in both dynamic and static conditions. The whole process of this investigation was repeated three times, and after obtaining all required information, the average width of the wound was determined at each time point.

### 2.4 Data collection

The culture flasks were placed inside the incubator after completing the wounding of the cells. Then, for a 48-h period, the wound width was observed and captured at every 2-h interval. Digital light microscopy (Olympus, UK) was used to capture the image of the cells. Images were processed using ImageJ imaging software to determine the distance between the wound edges. Again, for each cultured flask, eight horizontal lines were drawn to mark the wound edges. At a scratch width of 300 μm ±10–30 μm at 0 min, measurements were performed to distinguish the separations between the crossing points of the lines with wound edges. Different graphs were plotted between the rate of wound closure against time needed for that closure to determine the impact of TGF-β3 in bone cells’ wound healing against control or without treatment conditions (Supplementary Figure S2).

### 2.5 Statistical analysis

Mean % of wound remained open was tested for normality using a Kolmogorov Smirnov test. Results that showed normal distribution (*p* > 0.05) were analyzed using SPSS via a Oneway Analysis of Variance (ANOVA) followed by a *post hoc* Bonferroni test. Kruskal-Wallis test and serial Mann Whitney tests were used for non-normally distributed results (*p* < 0.05). Statistical tests were performed such that a *p-*value of <0.05 was considered as indicating a significant difference. There was some variability in initial wound widths therefore, % wound closure was normalized.

## 3 Results

### 3.1 Effects of static condition on cultured human bone cell monolayers’ wound closure

**Figure 1:**
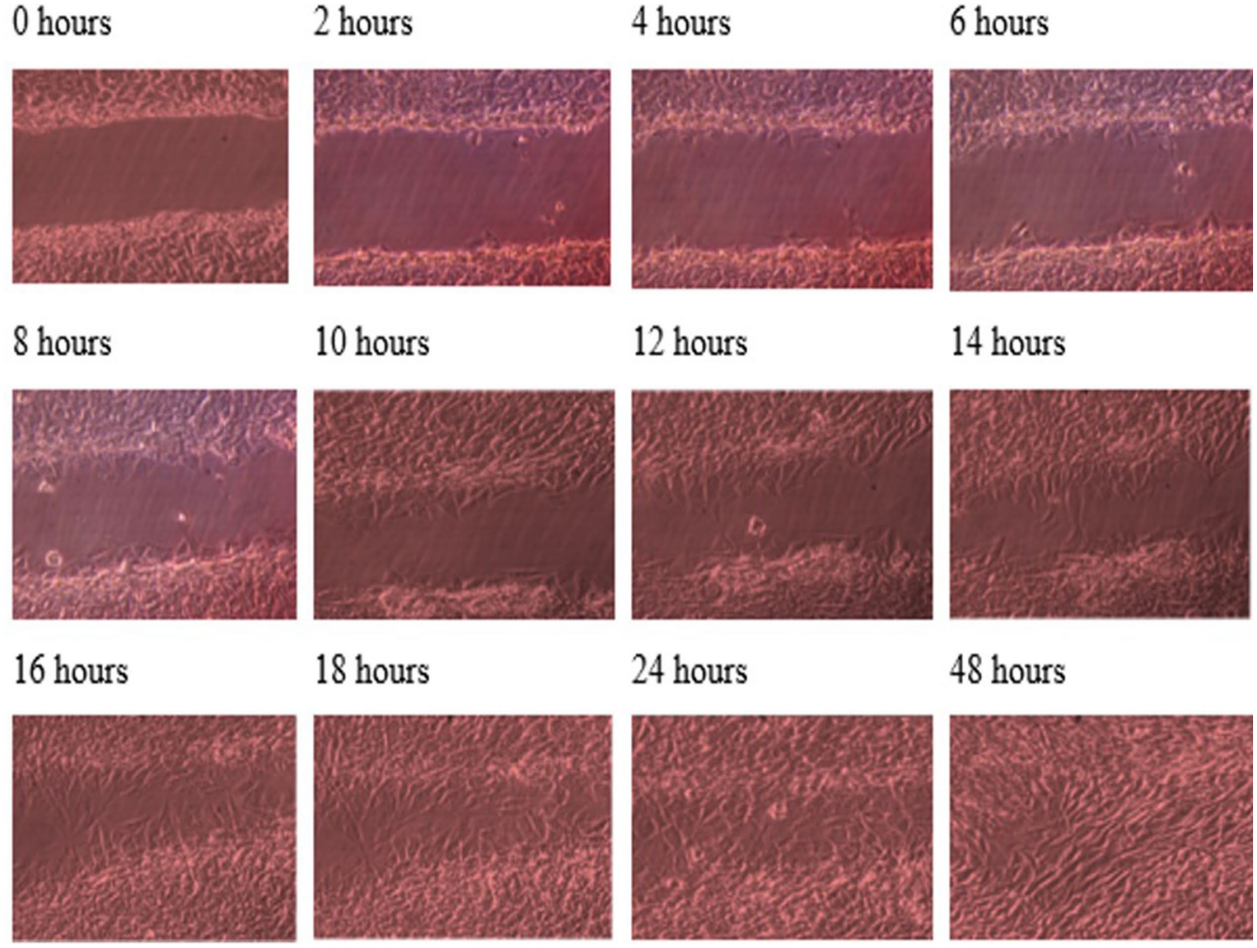
Photomicrographs of the wound healing process for the MG63 bone cell monolayers treated with TGF-β3 in static condition for the period of 48 h (Scale bar = 300 μm).

**Figure 2:**
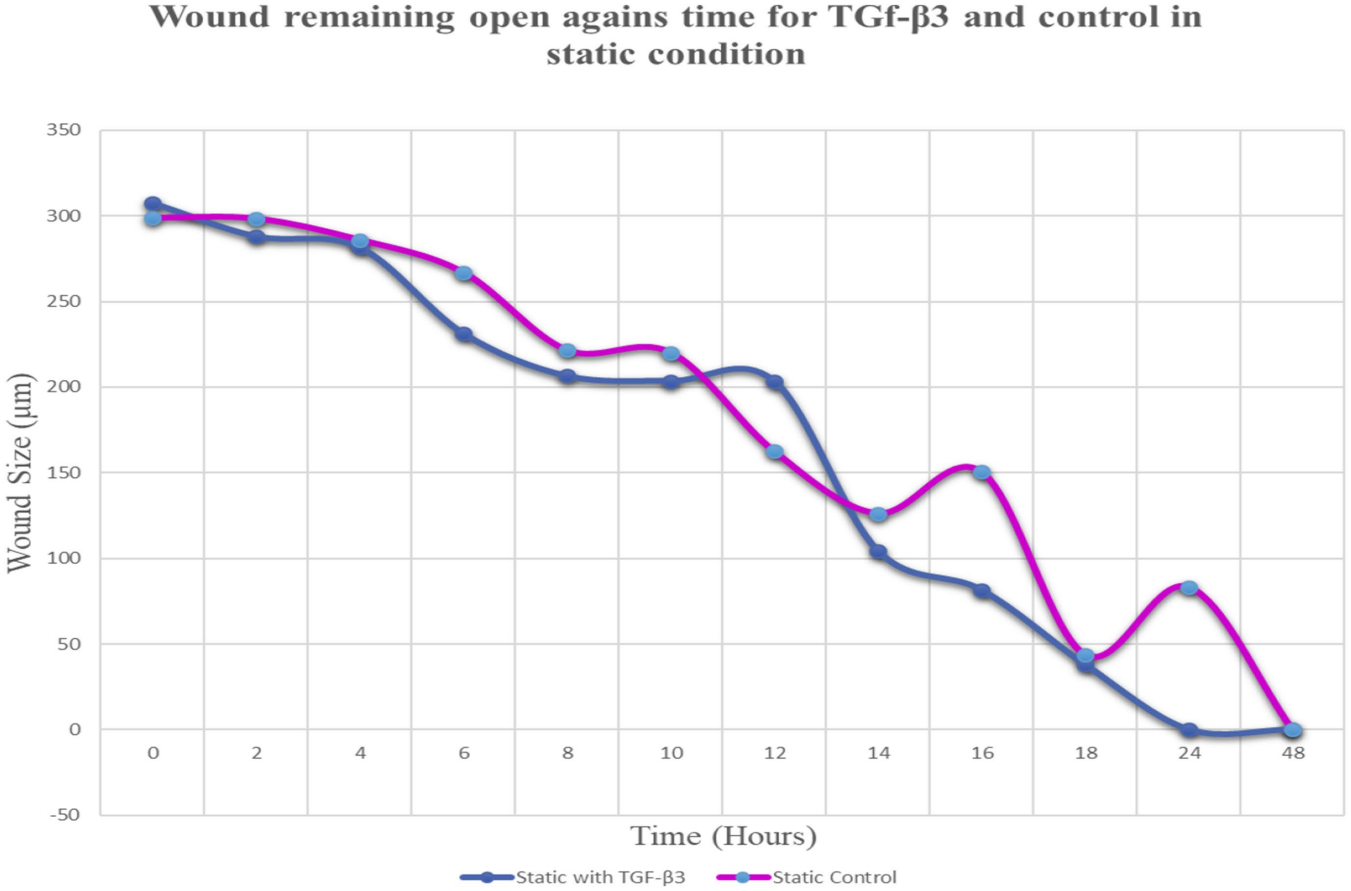
Graph of normalized the mean wound remaining open against time for wounded bone cell monolayers treated with TGF-β3 and control in static condition.

Throughout this experiment, the progress of wound closure for 48 h was investigated in static condition for both control (untreated) wounded bone cells and treated human bone cells (MG-63) with TGF-β3. During the investigation, wound closure in both cases was observed at an interval of 2 h; it was found that shortly after 24 h, wound healing in the culture flasks with wounded bone cells that were treated with TGF-β3 was more rapid than that in control flasks. Again, after 12 h of healing process, it was observed that cells on both sides of the wounds were extended to associate with other cells, forming bridges. Along the bridges, the cells were found to have moved and created a gap from the edge of the wound, which was adjusted to restore the structure of the cells. Those cells were extended in such a way that they could cover the gap created, due to the migration and elongation of the cells at the sites of the wound on the bone cells. It was observed that the wound sites were completely covered with cells after 24 h (*p* < 0.01). The extension of the cells, and their actions in bridging the gap created and the wound healing process, were observed even after 48 h. From the results, it was determined that in a cell-cultured environment, the wound healing process was most certainly affected by the TGF-β3, and the wound closure process was apparently speeded up due to the presence of TGF-β3 in the static condition. From the statistical analysis, it was found that the wound closure rate after 24 h was quicker when cells were treated with TGF-β3, compared to untreated cells or the control in the static condition (p < 0.01) (Table 1 and Figures 1, 2).

**Table 1:** Statistical analysis for mean of wound remaining open against time for wounded monolayers treated with TGF-β3 and un-treated as control in the statics condition.

**Wound closure time (Hrs)**											
	0	2	4	6	8	10	12	14	16	18	24
**Mean of wound remining open ± SD**											
**Control**	298.9 ± 21.4	286.9 ± 24.7	280.6 ± 43.6	267.1 ± 31.6	221.6 ± 30.7	220.2 ± 38.6	162.2 ± 7.5	126.5.19.1	150.5 ± 9.3	116.5 ± 31.8	83.2 ± 25
**TGF-β3**	307.6 ± 22.2	288.1 ± 14.8	281.7 ± 18.8	231.4 ± 12.9	206.6 ± 54.3	203.6 ± 54.3	165.4 ± 31.2	104.4 ± 1.8	81.5 ± 16.7	75.9 ± 36.7	0.00 ± 0.00

### 3.2 Effects of dynamic condition on cultured human bone cell monolayers’ wound closure

**Figure 3:**
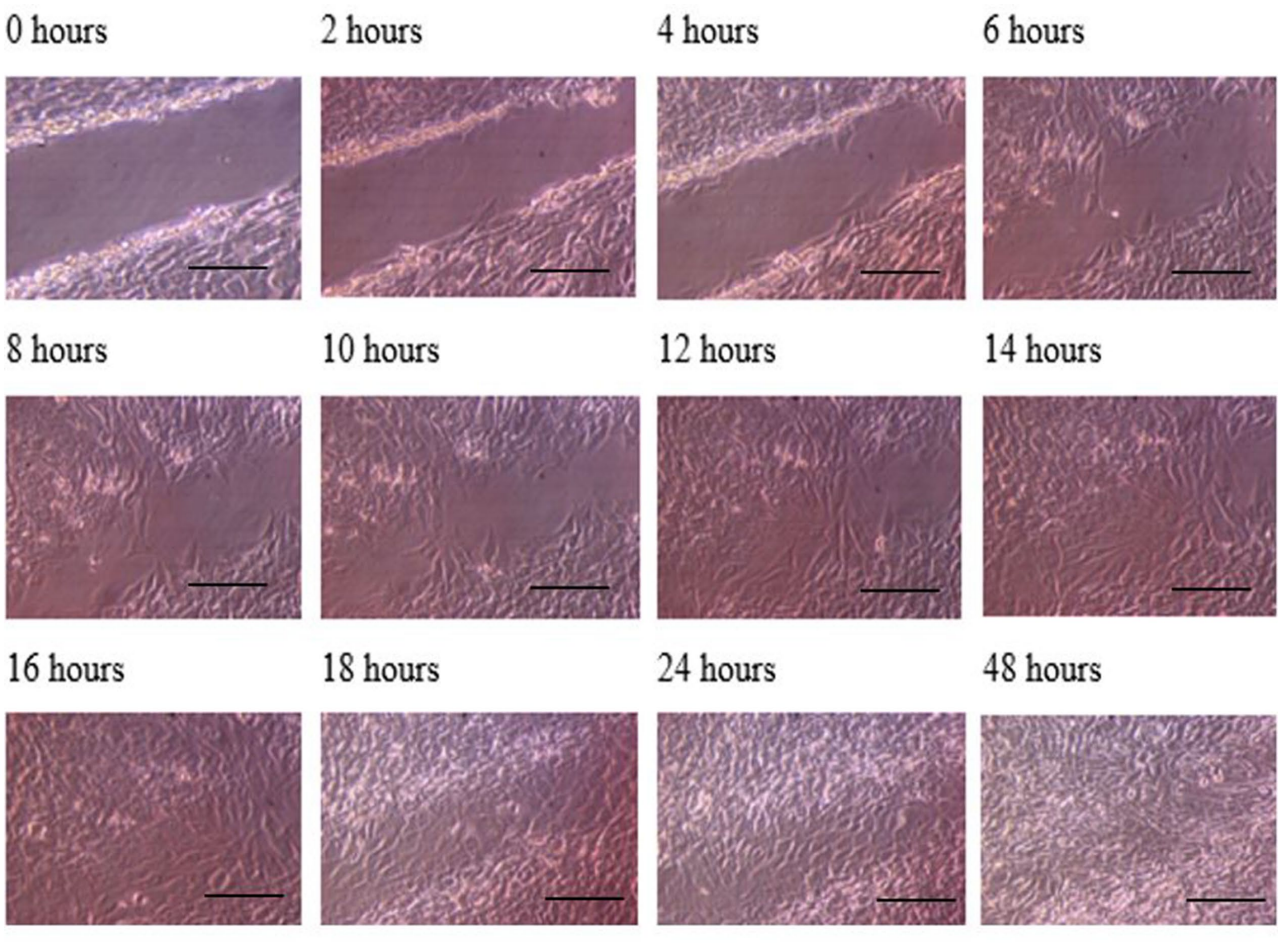
Photomicrographs of the wound healing process for the MG63 bone cell monolayers treated with TGF-β3 in Dynamic condition for the period of 48 h (Scale bar = 300 μm).

**Figure 4:**
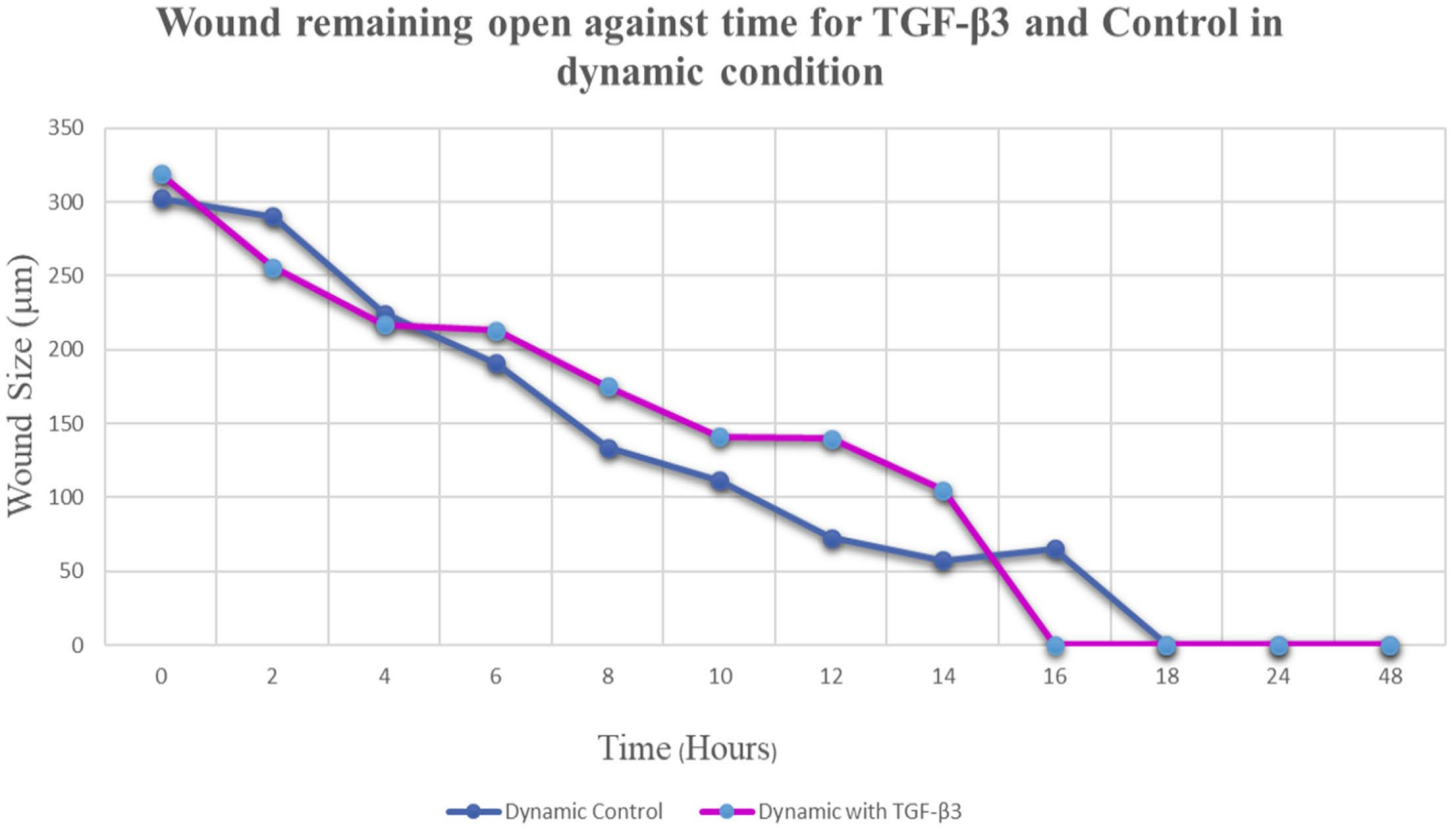
Graph of normalized the mean wound remaining open against time for wounded bone cell monolayers treated with TGF-β3 and control in the hydro dynamic condition.

The progress of wound closure for 48 h was investigated in the dynamic condition for both control (untreated) wounded bone cells and human bone cells (MG-63) treated with TGF-β3. During the investigation, wound closure in both cases was observed at an interval of 2 h, and it was found that shortly after 4 h, wound closure in the culture flasks with wounded bone cells that were treated with TGF-β3 occurred more rapidly than that in control flasks (*p* < 0.05). Again, after 8 h of healing process, it was observed that cells on both sides of the wounds were extended to be associated with other cells, forming bridges. Along the bridges, the cells were found to have moved and created a gap from the edge of the wound, which was adjusted to restore the structure of the cells. Those cells were extended in such a manner that they could cover the gap created due to the migration and elongation of the cells at the sites of the wound on bone cells. It was observed that the wound sites were completely covered with cells after 16 h (*p* < 0.01). After 16 h of the wound healing process, a high wound closure rate was found for each of the cells that were treated with TGF-β3 (*p* < 0.01). Moreover, it was found that wounded cells in the control or untreated flasks were healed after 18 h of the wound healing process (*p* < 0.01). The extension of the cells and their actions in bridging the gap created and the wound healing process were observed, even after 48 h. From the results, it was found that in a cell cultured environment the wound healing process was most certainly affected by the TGF-β3, and the wound closure process was apparently speeded up due to the shear stress force that occurred in the hydrodynamic condition (*p* < 0.001). From the statistical analysis, it was found that due to the shear stress in the hydrodynamic condition, the wound closure rate after 24 h was more rapid in the dynamic condition when compared to the static condition; this was true both for cells treated with TGF-β3 and untreated or control (*p* < 0.001). Furthermore, to standardize the results, the mean and standard deviation of the time that the wound remained open was calculated for both TGF-β3 and untreated or control for MG-63 human bone cells in the dynamic condition (*p* < 0.01) (Table 2 and Figures 3, 4).

**Table 2:** Statistical analysis for mean of wound remaining open against time for wounded monolayers treated with TGF-β3 and untreated as control in the dynamic condition.

**Wound closure time (Hrs)**											
	0	2	4	6	8	10	12	14	16	18	24
**Mean of wound remining open ± SD**											
**Control**	302.4 ± 14.8	290.2 ± 27.7	224.2 ± 55.6	190.9 ± 41.3	133.9 ± 51	111.3 ± 40.2	72.8 ± 28	57.4 ± 11.4	65.1 ± 8.2	0.00 ± 0.00	0.00 ± 0.00
**TGF-β3**	319.0 ± 31.5	225.8 ± 34.9	216.0 ± 50.3	213.1 ± 31.9	175.1 ± 32.8	140.9 ± 44.8	139.9 ± 33.7	104.9 ± 24.3	0.00 ± 0.00	0.00 ± 0.00	0.00 ± 0.00

### 3.3 Effect of static and dynamic conditions on scarless tissue

**Figure 5:**
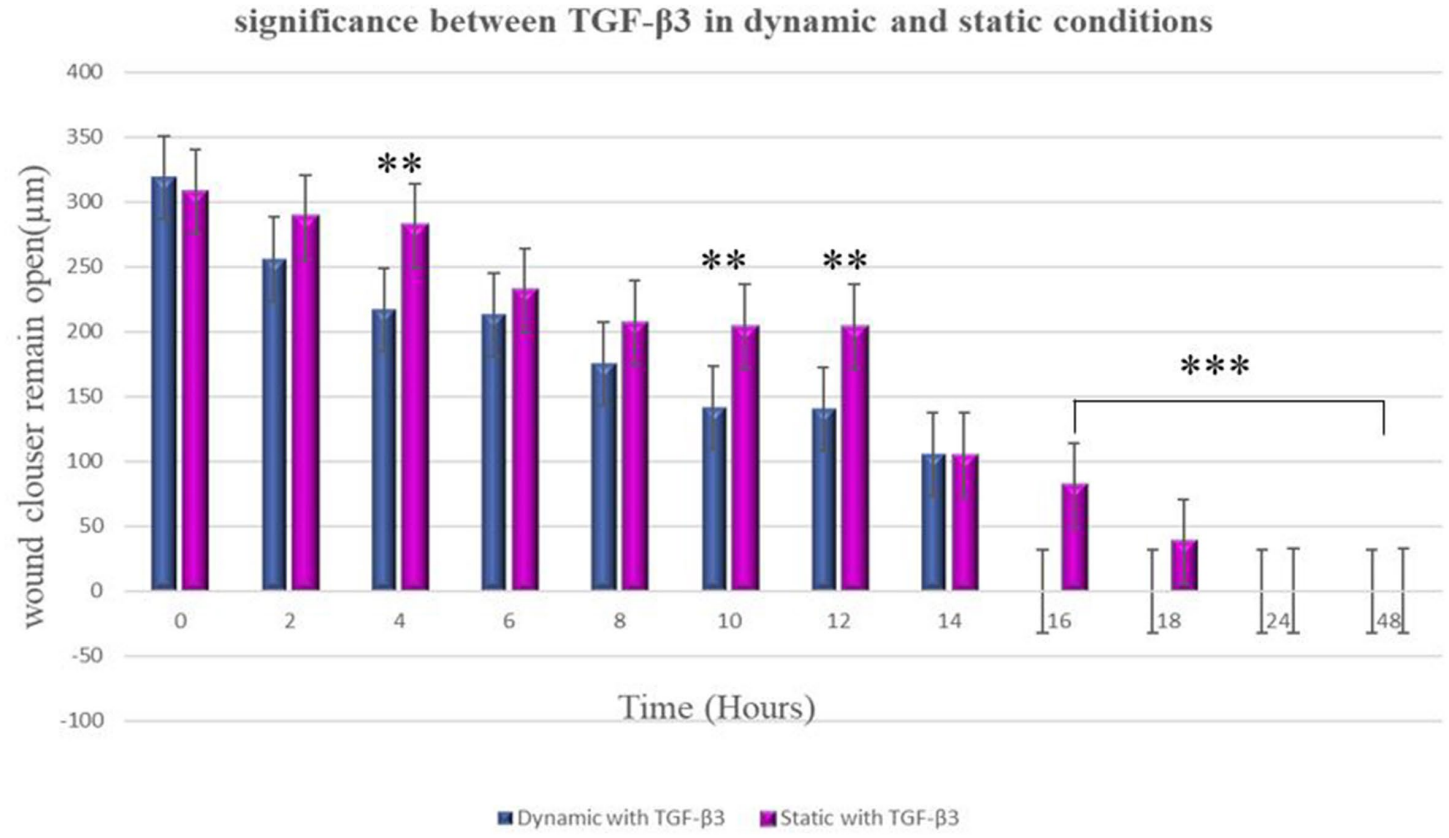
Significance differences between TGF-β3 in dynamic and static conditions. Two factors that have significant difference from one another noted by an asterisk (*) whereas *p < 0.05, **p < 0.01, and ***p < 0.001. Error bars represent standard deviation.

In the static condition, the wound of the bone cells healed after 24 h, and most of the cells aligned in one direction, confirming the formation of scar tissue. On the other hand, in the dynamic state, a significant difference was observed in terms of cell directions. Some cells were aligned in one direction, which confirms scar tissue formation, while other cells appeared in different directions. Therefore, it can be confirmed that less scar tissue formation occurs in dynamic conditions during the wound healing process (Figure 5). It is worth to know that MG63 bone cell monolayers treated with TGF-β3 in Dynamic condition formed bridge at 6 h after treatment while in static condition bridge formed at 14 h.

## 4 Discussion

This study was intended to demonstrate that TGF-β3, along with the synergistic effect of hydrodynamic shear stress, plays a significant role in wound healing of the bone cell monolayer.

Shear stress is essential for normal and abnormal physiology and various common physiological processes such as particle flow and blood flow produce shear stress that can trigger cellular behavior. On the other hand, shear stress has obvious roles in some normal activities within animal and human body such as wound healing and cancer. The fluid velocity and shear stress within bone are affected by various loading parameters (10). Extensive literature search showed that bone cells can go under high and low fluid shear stress (FSS) as well as long-term responses by cell monolayers. High FSS ranging between 0.5 and 2 Pa have been extensively reported to impact osteoblasts *in vitro*, such as changes in biochemical factors and gene expression (11, 12). On the other hand, osteoblasts unlike osteocytes might be more commonly subjected to low FSS. Osteoblast responses to low FSS below 0.5 Pa have also been reported (13–15). Finally, for long-term responses by cell monolayers which is more relevant to our study Morris et al. (16) cultured MLO-A5 osteoblasts in commercially available PPFC for 10 days while applying fairly high intermittent unidirectional fluid flow (0.8 Pa). We also looked at normal physiological blood flow in humans which generates a range of shear stress of 0.1–9.5 Pa (17–19). However, the parameter we used in this study is an average between high and low FSS.

TGF-β3 is one of the main regulators of wound healing and scar formation. It modulates the scar-forming processes, such as collagen synthesis and deposition, as well as tissue fibrosis regulation, via different molecular and cellular pathways. Due to this, it is essential to understand the impact of TGF-β growth factors on the reaction of wound closure and wound healing of bone cells.

Various isomers of TGF-β proteins have already been introduced to clinical trials. TGF-βs have been shown to be useful in many treatments such as wounds with impaired healing, fractures, mucositis, ischemia–reperfusion injuries, and autoimmune disease (20). Also, expression of TGF-β in some diseases such as glomerulonephritis, pulmonary fibrosis and keloids has been shown to be responsible for accumulation of detrimental scar tissue.

Application of active TGF-β2 for treatment of chronic progressive multiple sclerosis resulted in anemia and reversible nephrotoxicity in number of patients with no change in expanded disability status 9 (21)). On the opposite side, murine experimental allergic encephalomyelitis was improved by using myelin basic protein-activated T cells transduced with latent TGF-β1 (22). While application of adenoviral vectors of TGF-β or TGF-β antagonists is being widely used in animal models (23–27). Also, application of active TGF-β has been reported to have an effect for venous leg ulcers (28) and finally, TGF-β3 showed positive effect in treating chemotherapy-induced oral mucositis in patients with lymphomas and solid tumors (29).

In the current study, after creating wounds (by scratching) on the monolayers of bone cells, the mean wound closure was found to remain open even after being kept in the incubator for 2 h, which may indicate that the different rates for wound closure and the recovery of bone cell wounds largely rely on varying aspects. The wound closure rate was much more rapid in the case of the culture flasks which were treated with TGF-β3 compared to the untreated or control culture flasks. The wound closure rate was found to be notably improved in the case of TGF-β3 treatments. However, no significant difference was found in terms of the rate of wound closure in the case of the culture flasks which were treated with TGF-β3 compared to the untreated or control culture flasks in the dynamic condition after 24 h in culture. Wound closure rates, in both cases, seemed to be increased, and then slowly became equal to the wound closure rate for each condition in the case of the dynamic condition because, in this case, bone cells were stimulated by the hydrodynamic shear force, which caused them to communicate with each other in an efficient and more rapid manner. Alternatively, the wound length for the culture flasks with TGF-β3, as found in the static condition, showed a significant increase in the rate of wound closure compared to that of control culture flasks that were untreated. By increasing the healing time for more than 24 h, the healing rate was found to be increased in both static and dynamic conditions for the cells in culture flasks treated with TGF-β3.

Based on the current findings, it is crucial to understand the mechanism behind these more rapid and efficient responses of the cell monolayers of culture flasks with TGF-β3 when compared to control. For this, the role of cytokines needs to be understood, as it is a powerful and significant biomolecule. Cytokines manage cell capacities and adopt various activities throughout the initiation and recovery of infection (30, 31). Moreover, the superfamily of transforming growth factors consists of cytokines, and these cytokines are normally responsible for various cellular functions and activities such as cell relocation, multiplication of cells, apoptosis, and cell attachment. The first introduction of TGF-β occurred in 1978 (32), and it was developed with all different phenotypes in a passive structure (33), which means that nearly all modes of administration in various types of tissue were investigated. Those studies were more focused on the impact of TGF-β on bone and did not focus on the effects of TGF-β on repairing bone injury or wounds. Studies investigated the impact of TGF-β on cell changes as well as their effect when administered to film proteins, while other studies showed that TGF-β impacts the initiation of cell expansion, detachment, grip, enactment, apoptosis, and movement (34–38). According to Huang et al. (39), these growth factors can control cell improvement and advancement in a powerful manner. Feng and Zuo (40) demonstrated the ability of TGF-β2 in bone repair by employing a display with TGF-β2 for more than 2.5 months in a case of tibial deformations and found that TGF-β2 can positively and significantly repair bone. In the case of wound healing, cells behave differently in different conditions. Cell connections typically communicate using ligands, through film receptors of adjacent cells, and cell–cell flagging is a vital activity in cell growth and expansion (41). Based on the current findings, the wounded bone cells in the culture flasks which were treated with TGF-β3 showed a rapid decrease in wound width within 24 h of the recuperation of the wounds, which can be linked with the activity of TGF-β3, where emission of the extracellular matrix (ECM) particles was expanded by TGF-β3 at the wound edges. This process functions by grasping the cells using the integrin ECM at the wound edge, which helps the movement at the site of the wound. Furthermore, shear force was applied through the cells; this shear force is considered as an essential prevention strategy of bone wound healing, as it fills the wound site with a large number of bone cells and this, ultimately, enables the ECM over the wound surface to move and multiply cells into the wound site (41). A study by Sefat et al. (9) demonstrated that the TGF-β3 usually does not animate the bonding between bone and ECM. Refined bone cells and TGF-β3 demonstrated that the extent of cell surface association was reduced in the static condition. Therefore, TGF-β3 showed a reduced wound healing in the static condition compared to that under dynamic conditions. This implies that the static condition, in this case, slows down the control of the process of cell movement and multiplication. In most cases, for the scratched cells, the wound healing process started after 2 h since this process is responsive to stress or pressure shock on the cell. Similar findings were reported in various other studies related to keratinocytes and chondrocytes (42–44). Moreover, to target explicit types of cells, development factors can also be associated with certain capacities such as cell separation, multiplication, and movement. A study by Wan et al., conducted with a comparative methodology and various elements to enhance *in vitro* osteogenic articulation with the use of a combination of different cytokines, such as bone morphogenic protein (BMP-2) and TGF-β1, found that the superfamily of transforming growth factors, including TGF-β1, TGF-β2, and TGF-β3, expressively complete their explicit activities in the new bone development (45). A study by Sefat et al. investigated a mix of different growth factors, such as TGF-β1 and TGF-β2; TGF-β2 and TGF-β3; TGF-β3 and TGF-β1; or TGF-β1, TGF-β2, and TGF-β3; to determine their impacts on wound healing. From the study, it was determined that, in the case of wound healing, TGF-β3 plays a crucial role in bone repair and provides a better rate of wound healing than any other growth factors used independently or in combination. Therefore, it can be stated that TGF-β3 has a significant impact on the bone cell (9). Similar to these findings, the current study illustrated that the wound healing process is improved and shortened by the presence of TGF-β3. Therefore, it can be confirmed that TGF-β3 facilitates cell–cell communication and has a significant impact on the behavior of bone cells. Furthermore, cell migration was found to be facilitated by shear force created in the dynamic condition, which promotes and increases the wound closure rate.

## 5 Conclusion

The main finding of our study is that the synergistic effect of TGF-β3 and the hydrodynamic shear force condition play a significant role in bone cells’ wound closure. TGF-β3 can be used as a successful and effective therapeutic method for wound healing. These findings confirmed that when a culture flask was treated with TGF-β3, bridge creation occurs at the site of the wound after about 8 h, whereas complete healing of the wound occurs after 16 h. This finding implies that bridge formation is an important aspect to be considered in the wound healing process and that, with the help of bridging, the wound closure rate increases significantly. In the case of static conditions, TGF-β3 treatments showed an efficient result; the wound closure rate was more rapid than that found in the control flask. Therefore, it can be suggested that wound closure can be postponed in the static condition while, in the dynamic condition, the wound healing process became more rapid in a cultured cell environment. Moreover, TGF-β3 treatment demonstrated greater effectiveness in wound healing than the control flask under both static and dynamic conditions. On the other hand, wound closure depends on various aspects, such as the migratory phenotype and proliferation of the cells, and elongations of the cells in the presence of TGF-β3. Our future aims to investigate the migration behavior in microfluidics-based wound healing assay. Microfluidics offers high controllability in cellular microenvironments while consuming low quantities of samples and reagents. Indeed, microfluidic cell culture provides adequate flexibility to customize chips in precisely defined geometries, enabling culturing and performing different analytic investigations on the same platform.

## Data availability statement

The original contributions presented in the study are included in the article/Supplementary material, further inquiries can be directed to the corresponding authors.

## Author contributions

HA-A: Conceptualization, Data curation, Formal analysis, Methodology, Writing – original draft, Writing – review & editing. LD: Data curation, Visualization, Writing – review & editing. SK: Conceptualization, Supervision, Visualization, Writing – review & editing. CT-B: Project administration, Visualization, Writing – review & editing. SD: Writing – review & editing. MS: Data curation, Project administration, Validation, Writing – original draft, Writing – review & editing. FS: Conceptualization, Data curation, Investigation, Methodology, Project administration, Supervision, Validation, Visualization, Writing – original draft, Writing – review & editing.
