# Supplementary material for: Effect of TGF-β3 on wound healing of bone cell monolayer in static and hydrodynamic shear stress conditions
Source: Front Med (Lausanne). 2024 Apr 24;11:1328466. doi: 10.3389/fmed.2024.1328466 (PMC11076756; doi:10.3389/fmed.2024.1328466)
Supplement: Supplementary file 1 [file Data_Sheet_1.docx]

**Effect of TGF-β3 On Wound Healing of Bone Cell Monolayer in Static and Hydrodynamic Shear Stress Conditions**

Hawra Al-Attar^1^, Laila A. Damiati^2*^, Saeed Heidari Keshel^3^, Cristina Tuinea-Bobe^1,4^, Samar Damiati^5^, Morvarid Saeinasab^1^, Farshid Sefat^1, 4*^

^1^ Department of Biomedical and Electronics Engineering, School of Engineering, University of Bradford, Bradford, UK

^2^ Department of Biological Sciences, Collage of Science, University of Jeddah, Jeddah, Saudi Arabia

^3^ Department of Tissue Engineering and Applied Cell Sciences, School of Advanced Technologies in Medicine, Shahid Beheshti University of Medical Sciences, Tehran, Iran

^4^ Interdisciplinary Research Centre in Polymer Science & Technology (IRC Polymer), University of Bradford, Bradford, UK

^5^ Department of Chemistry, College of Sciences, University of Sharjah, Sharjah, United Arab Emirates

***Corresponding Author:**

**Farshid Sefat,** [F.Sefat1@Bradford.ac.uk](mailto:F.Sefat1@Bradford.ac.uk)

**Laila A. Damiati,** Ladamiati@uj.edu.sa


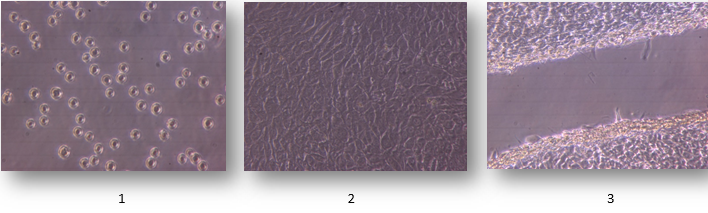


Supplement Figure 1: Growth of human bone cells (MG63) in culture flask: (1) after cell passage (2) confluence cell monolayer (after 48 hrs. in culture) and (3) wounded cells ready for the test; X 100 magnification.


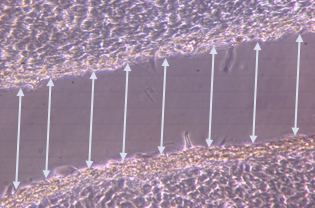


Supplement Figure 2: Measurement of wound closure width on cultured MG63 bone cell monolayer using ImageJ software
